# Gut Microbiota and Risk of Persistent Nonalcoholic Fatty Liver Diseases

**DOI:** 10.3390/jcm8081089

**Published:** 2019-07-24

**Authors:** Han-Na Kim, Eun-Jeong Joo, Hae Suk Cheong, Yejin Kim, Hyung-Lae Kim, Hocheol Shin, Yoosoo Chang, Seungho Ryu

**Affiliations:** 1 Medical Research Institute, Kangbuk Samsung Hospital, Sungkyunkwan University, School of Medicine, Seoul 03181, Korea; 2 Center for Cohort Studies, Total Healthcare Center, Kangbuk Samsung Hospital, Sungkyunkwan University, School of Medicine, Seoul 04514, Korea; 3 Department of Clinical Research Design & Evaluation, SAIHST, Sungkyunkwan University, Seoul 06351, Korea; 4 Division of Infectious Diseases, Department of Internal Medicine, Kangbuk Samsung Hospital, Sungkyunkwan University, School of Medicine, Seoul 03181, Korea; 5 Department of Biochemistry, College of Medicine, Ewha Womans University, Seoul 07804, Korea; 6 Department of Family Medicine, Kangbuk Samsung Hospital, Sungkyunkwan University School of Medicine, Seoul 03181, Korea; 7 Department of Occupational and Environmental Medicine, Kangbuk Samsung Hospital, Sungkyunkwan University, School of Medicine, Seoul 04514, Korea

**Keywords:** NAFLD, microbiota, 16S rRNA

## Abstract

Gut dysbiosis is regarded as a pathogenetic factor of nonalcoholic fatty liver disease (NAFLD), but its role in NAFLD persistence is unknown. We investigated the influence of the gut microbiota on persistent NAFLD. This cohort study included 766 subjects with 16S ribosomal RNA (rRNA) gene sequencing data from fecal samples at baseline who underwent repeated health check-up examinations. Fatty liver was determined using ultrasound at baseline and follow-up. Participants were categorized into four groups: none (control), developed, regressed, or persistent NAFLD. The persistent NAFLD group had lower richness compared with the control group. Significant differences were also found in both non-phylogenic and phylogenic beta diversity measures according to NAFLD persistence. Pairwise comparisons indicated that taxa abundance mainly differed between the control and persistent NAFLD groups. A relative high abundance of Fusobacteria and low abundance of genera *Oscillospira* and *Ruminococcus* of the family Ruminococcaceae and genus *Coprococcus* of the family Lachnospiraceae were found in the persistent NAFLD group. Based on the functional predictions, pathways related to primary and secondary bile acid biosynthesis were highly detected in the persistent NAFLD group compared with the control group. These findings support that the composition of the gut microbiome associated with dysregulation of bile acid biosynthetic pathways may contribute to the persistence of NAFLD. This is the first cohort study to demonstrate the influence of microbiota on persistent NAFLD. Our findings may help identify potential targets for therapeutic intervention in NAFLD.

## 1. Introduction

Nonalcoholic fatty liver disease (NAFLD) comprises a spectrum of liver diseases ranging from simple steatosis to nonalcoholic steatohepatitis (NASH) that can progress to fibrosis, cirrhosis, liver failure, or hepatocellular carcinoma in a subgroup of patients [1,2]. The prevalence of NAFLD rose globally, making NAFLD the most common liver disease worldwide with the estimated global prevalence of 25.2% and a significant public health burden [3,4,5]. NAFLD is also considered a multisystem disease that is closely associated with various extrahepatic diseases, including type 2 diabetes mellitus, metabolic syndrome, chronic kidney disease, and cardiovascular disease (CVD) [6,7,8].

NAFLD is a reversible condition and fatty liver status can change over time [9,10]. Several studies suggested that persistent NAFLD is associated with an increased risk of incident diabetes or subclinical atherosclerosis [11,12]. In a large cohort study, patients with persistent NAFLD had a higher risk of subclinical carotid atherosclerosis development compared with those without NAFLD that was mediated by metabolic risk factors; this highlights the importance of persistent NAFLD as a risk factor for metabolic diseases and atherosclerotic CVD [11]. Identifying the underlying mechanisms for persistent NAFLD could be useful for developing new therapeutic targets.

The gut microbiota is now known to be an important factor in regulating homeostasis of the gastrointestinal tract and affecting the immune system, as well as the metabolism of the host [13]. Recently, the role of gut microbiota was demonstrated in the development of NAFLD and its progression to NASH [14]. Several previous cross-sectional studies revealed an association between the gut microbiota and the presence of NAFLD or NASH in healthy controls and patients with biopsy-proven NAFLD who were referred to hepatology clinics for persistently elevated liver enzymes [15,16,17,18]. Until now, however, the effect of the microbiome on persistent NAFLD over time was not evaluated and remains unknown. In this large cohort study, we investigated the influence of the microbiota on persistent NAFLD. To define persistent NAFLD, we classified participants into four groups based on NAFLD status at baseline and follow-up: (1) none (control), those without fatty liver both at baseline and follow-up; (2) developed, those without fatty liver at baseline but with fatty liver at follow-up; (3) regressed, those with fatty liver at baseline but without fatty liver at follow-up; and (4) persistent, those with fatty liver both at baseline and follow-up.

## 2. Methods

### 2.1. Study Population and Study Design

**Figure 1:**
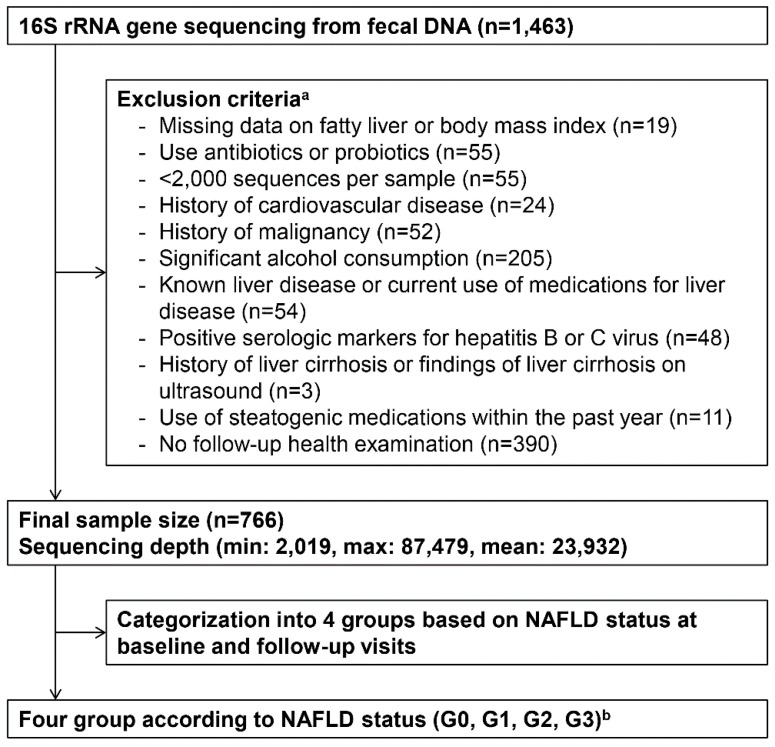
Enrolment of subjects. Inclusion criteria: 1463 Korean adults aged 23 to 78 years who agreed to participate in this study and provided stool samples at Kangbuk Samsung Hospital Healthcare Screening Center out of those who underwent annual or biennial examinations during the study period between June 2014 and September 2014. ^a^ Some individuals met several exclusion criteria. ^b^ G0, none (control), patients without fatty liver both at baseline and follow-up; G1, developed, patients without fatty liver at baseline but with fatty liver at follow-up; G2, regressed, patients with fatty liver at baseline but without fatty liver at follow-up; G3, persistent, patients with fatty liver both at baseline and follow-up.

The Kangbuk Samsung Health Study is a cohort study of Korean men and women who undergo comprehensive annual or biennial examinations at the Kangbuk Samsung Hospital Healthcare Screening Center in South Korea [19]. The present study included 1463 Korean adults aged 23 to 78 years, who agreed to participate in this study and provided stool samples at Kangbuk Samsung Hospital Healthcare Screening Center. These participants were recruited from those who underwent annual or biennial examinations during the study period between June 2014 and September 2014. Written informed consent was obtained from all individual participants included in this study. The fecal samples were obtained once only at the baseline and not at follow-up visits. Participants who met any of the exclusion criteria as described were not included in the analysis (Figure 1). We excluded 697 subjects based on the following criteria: the use of antibiotics within six weeks prior to enrolment or probiotics within six weeks prior to enrolment (*n* = 55); samples with fewer than 2000 sequences per sample (see below for further details) (*n* = 19); history of CVD (*n* = 24); history of malignancy (*n* = 52); significant alcohol consumption defined as alcohol intake of ≥30 g/day for men or ≥20 g/day for women [3] (*n* = 205); known liver disease or current use of medications for liver disease (*n* = 54); positive serologic markers for hepatitis B or C virus (*n* = 48); history of liver cirrhosis or findings of liver cirrhosis on ultrasound (US) (*n* = 3); and use of steatogenic medications within the past year, such as valproate, amiodarone, methotrexate, tamoxifen, or corticosteroids (*n* = 11) [3]. Because the change in NAFLD status over time was the main outcome of this study, participants with no follow-up visit by 31 December 2017, were also excluded (*n* = 390). Some individuals met more than one exclusion criterion, and a total of 766 participants were included in the final analysis.

Ethical approval for the phenotype, genotype, and microbiota studies within the Kangbuk Samsung Cohort Study was provided by the institutional review boards of Kangbuk Samsung Hospital (KBSMC 2013-01-245-12). Written consent was obtained from all participants after the nature and possible consequences of the studies were explained. All applicable institutional and governmental regulations concerning the ethical use of human volunteers were followed during this research. The research was carried out in accordance with the Declaration of Helsinki.

### 2.2. Data Collection

Data on medical history, medication use, and health-related behaviors were collected through a self-administered questionnaire, while the physical measurements, US, and biochemical parameters were measured by trained staff during the health examinations. The questionnaire asked about the frequency of alcohol drinking and the amount of alcohol consumed per drinking day, recorded in standard units. The average alcohol intake per day was calculated using the frequency and the amount of alcohol consumed per drinking day. Excessive alcohol intake was defined using average alcohol intake [3,20]. Smoking status was categorized as never, former, or current smoker. Physical activity was assessed using the short form of the validated Korean version of the International Physical Activity Questionnaire [21] and was categorized as being inactive, being minimally active, or engaging in health-enhancing physical activity. Height and weight were measured by trained nurses. Obesity was defined as a body mass index (BMI) ≥25 kg/m^2^ according to Asian-specific criteria and is the same BMI cutoff for defining obesity in Korea [22]. Hypertension was defined as systolic blood pressure (BP) ≥140 mmHg, a diastolic BP ≥90 mmHg, or the use of antihypertensive medications. Diabetes mellitus was defined as a fasting serum glucose ≥126 mg/dL, HBA1c ≥6.5%, a self-report of a previous diagnosis, or use of blood glucose-lowering agents.

### 2.3. NAFLD and Group Definition

The diagnosis of fatty liver was based on abdominal US operated by experienced radiologists who were blinded to the aim of the present study, and was determined using standard criteria, including the presence of a diffuse increase of fine echoes in the liver parenchyma compared with kidney or spleen parenchyma, deep beam attenuation, and bright vessel walls [23]. Inter-observer and intra-observer reliability for fatty liver diagnosis were substantial (kappa statistic of 0.74) and excellent (kappa statistic of 0.94), respectively. NAFLD was defined as (1) the presence of fatty liver on the US, (2) the lack of excessive alcohol consumption (alcohol intake of <20 g/d for women and <30 g/d for men), and [3] other identifiable causes of hepatic fat accumulation (see exclusion criteria).

Based on the NAFLD status at both baseline and subsequent visits, we categorized patients into four groups as follows: Group 0 (G0): none (control), those without fatty liver both at baseline and subsequent follow-up; Group 1 (G1): developed, those without fatty liver at baseline but with fatty liver at follow-up; Group 2 (G2): regressed, those with fatty liver at baseline but without fatty liver at follow-up; and Group 3 (G3): persistent, those with fatty liver both at baseline and subsequent follow-up.

### 2.4. DNA Extraction from Fecal Samples and Sequencing of the Bacterial 16S Ribosomal RNA (rRNA) Gene

Fecal samples were collected at participants’ homes within 24 h before visiting the hospital. The container and stick for feces collection and instructions on how to collect and store feces were provided to participants in advance. They were instructed to defecate onto a stool collection sheet above the toilet water at home, to transfer a sample of stool into the container without preservative, and to store the sample in a −20 °C freezer immediately after sampling. The samples were immediately stored in a −70 °C freezer in the laboratory after the participants arrived at the hospital. DNA extraction from fecal samples was performed within one month of storage using the MOBio PowerSoil^®^ DNA Isolation Kit (MO BIO Laboratories, Carlsbad, CA, USA) according to the manufacturer’s instructions. Amplification and sequencing were performed for the analysis of bacterial communities, as described previously [24]. Genomic DNA was amplified using fusion primers targeting the variable V3 and V4 regions of the 16S rRNA gene with indexing barcodes. Samples were pooled for sequencing on the Illumina Miseq platform (Illumina, San Diego, CA, USA) according to the manufacturer’s instructions [25].

### 2.5. 16S rRNA Gene Compositional Analysis

The DADA2 [26] pipeline of the QIIME2 package (version 2018.08, https://qiime2.org) [27] was used to generate unique sequence variants by filtering low-quality samples and chimera. The amplicon sequence variants (ASVs) were produced by denoising with DADA2 and regarded as 100% operational taxonomic units (OTUs). For taxonomic analysis, taxonomy was assigned to the sequence variants using a pre-trained naïve Bayes classifier and the q2-feature-classifier plugin. This classifier was trained on the V3–V4 region containing genes from the Greengene 13_8 99% OTUs of the 16S rRNA gene sequences in the QIIME2 package.

### 2.6. Statistical Analysis

Basic statistical analyses were performed using RStudio (version 0.98.983, Boston, MA, USA). For diversity analysis, we rarefied the feature tables to 2000 sequences per sample by random subsampling in QIIME2. Alpha diversity was estimated using the actual number of different taxa observed in a sample (“observed ASVs”) and a phylogenetic diversity (PD) measurement, Faith’s PD, which considers phylogenetic differences between ASVs [28]. Alpha diversity was also measured with the Shannon index by accounting for evenness and richness. The Kruskal–Wallis test was used to estimate the median difference among all groups or pairwise groups. Beta diversity was used to analyze the dissimilarity among group membership and structure. Non-phylogenetic methods were used with Bray–Curtis dissimilarities [29] due to the abundance of data. Phylogenetic methods, including weighted and unweighted UniFrac distance [30], were also used for the abundance and the presence/absence data, respectively. Permutational multivariate analysis of variance (PERMANOVA, with 999 permutations) was used to test significance for the beta diversity among groups. The comparisons between the two groups within the four groups were measured using pairwise PERMANOVA. The resulting *p-*values were corrected for multiple comparisons using the Benjamini–Hochberg correction (false discovery rate (FDR), *q-*value) for both alpha and beta diversity analyses. A *q*-value <0.05 was considered statistically significant.

Significant differences in microbial taxa abundance between two groups were analyzed using the analysis of the composition of the microbiome (ANCOM) [31]. ANCOM compares the relative abundance of taxa among multiple groups by log-ratio of the abundance of each taxon to the abundance of all the remaining taxa one at a time. To adjust for confounding variables (age, sex, and BMI), we used the ANCOM2 code shared by the author from the original ANCOM paper [31], which can deal with covariates. The final significance was expressed in the empirical distribution of W from analyses for four groups. We also performed multiple ANCOM tests across the pairwise combinations separately, as well as the generalized linear models using MaAsLin packages [32]. To detect bacterial taxa with significantly different abundances between the control and NAFLD persistent groups, linear discriminant analysis (LDA) effect size (LEfSe) analysis was used according to the online protocol without adjusting for covariates (https://huttenhower.sph.harvard.edu/galaxy/) [33].

Additionally, microbial community function was evaluated by predictive metagenome analysis using Phylogenetic Investigation of Communities by Reconstruction of Unobserved States (PICRUSt) [34]. PICRUSt is a phylogeny-based computational tool that predicts the functional capacity of microbial communities by correlating the species present with the reference databases of microbial genomes. We performed PICRUSt with de novo variants according to a recent manual (https://github.com/LangilleLab/microbiome_helper/wiki/PICRUSt-Tutorial-with-de-novo-Variants). DADA2 variants were normalized by 16S rRNA copy number, and KEGG (Kyoto Encyclopedia of Genes and Genomes) orthologs (KOs) were predicted. Results that aggregated to level 3 of the KEGG analysis module were further explored with statistical analysis of taxonomic and functional profiles (STAMP) version 2.1.3 [35], using the multi-group analysis module. The post hoc test was used with Tukey–Kramer. The resulting *p*-values were corrected for multiple comparisons on the number of pathways using Bonferroni correction (*q-*value). The effect size >0.03 and *q*-value <0.05 were considered statistically significant for the PICRUSt results.

## 3. Results

### 3.1. Demographics of the Subjects

The baseline characteristics of enrolled subjects according to the NAFLD groups are presented in Table 1. Among the total 766 subjects, 453 (59.1%) were in G0, 40 (5.2%) were in G1, 35 (4.6%) were in G2, and 238 (31.1%) were in G3. Participants with persistent NAFLD were more likely to be older and male, have hypertension and diabetes, have higher BMI and worse profiles of all lipid levels, and be less physically active than those without NAFLD.

### 3.2. Overall Structure of the Fecal Bacterial Communities among NAFLD Groups: Alpha and Beta Diversity

**Figure 2:**
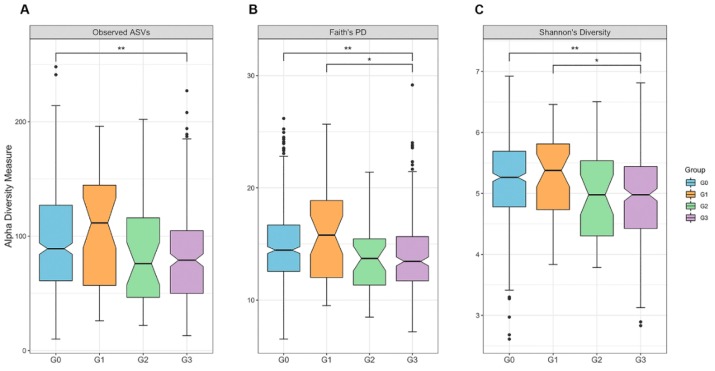
Alpha diversity among groups: (**A**) observed features (*p* = 1.9 × 10^−3^ for all groups, Kruskal–Wallis test), (**B**) phylogenetic diversity (Faith’s PD, *p* = 2.7 × 10^−4^ for all groups), (**C**) Shannon index (*p* = 3.2 × 10^−5^ for all group). * *q* < 0.05, ** *q* < 0.01 (pairwise Kruskal–Wallis test, Benjamini–Hochberg correction). The notched boxes indicate the interquartile range (IQR). The IQR is the 25th to 75th percentile. The median value is shown as a line within the box and the notch indicates the 95% confidence interval of the median. Whiskers extend to the most extreme value within 1.5 × IQR. Possible outliers are shown as dots.

The sequencing depth ranged from 2019 to 87,479 reads per sample (mean = 23,932) and 7270 features in 766 subjects. The alpha diversity of gut microbiota among the G0, G1, G2, and G3 groups showed statistically significant differences in the observed ASVs (*p* = 1.9 × 10^−3^), Faith’s PD (*p* = 2.7 × 10^−4^), and Shannon’s index (*p* = 3.2 × 10^−5^). In the Kruskal–Wallis pairwise test, there were differences between the G0 and G3 groups (*q* = 6.3 × 10^−4^; 4.8 × 10^−5^) and between the G1 and G3 groups (*q* = 3.8 × 10^−2^; 4.8 × 10^−5^) in both the Faith’s PD and the Shannon index, respectively. The G3 group had lower richness than the G0 and G1 groups, as shown in Figure 2 and Table S1 (Supplementary Materials).

**Figure 3:**
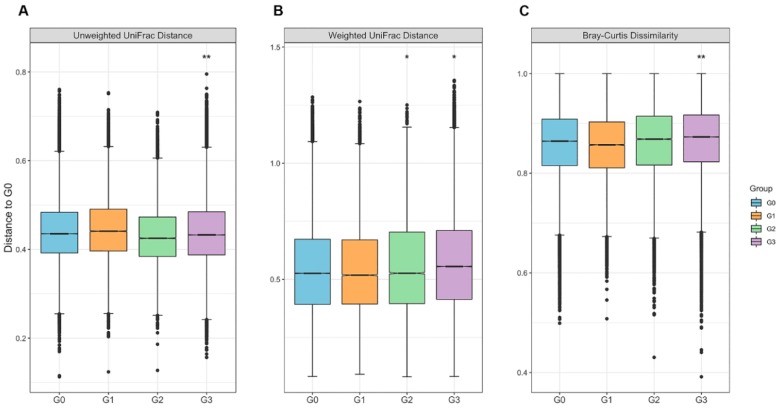
Beta diversity among groups: (**A**) unweighted UniFrac distance (*p* = 0.001 for all groups, permutational multivariate analysis of variance (PERMANOVA)), (**B**) weighted UniFrac distance (*p* = 0.003 for all groups, PERMANOVA), and (**C**) Bray–Curtis dissimilarity (*p* = 0.01 for all groups, PERMANOVA). The *y*-axes represent the distance of each group to the G0 group (baseline). The line in each box indicates the median of the data. The *p*-values among all groups were estimated using PERMANOVA with 999 permutations. The *q-*values were estimated using pairwise PERMANOVA (* *q* < 0.05, ** *q* < 0.01).

The beta diversity analysis indicates the extent of similarity and differences among microbial communities. To quantify the beta diversity, both non-phylogenic and phylogenic methods were used with Bray–Curtis dissimilarity and Unifrac distance, respectively (Figure 3). The Bray–Curtis dissimilarity showed differences across all four groups (*p* = 0.01, pseudo-F = 1.26, PERMANOVA). The results of the pairwise PERMANOVA demonstrated the significant community differences between the G0 and G3 groups (*q* = 0.006, pseudo-F = 1.98). In the phylogenic distance indices, both the weighted and unweighted Unifrac distances also indicated distinct community compositions across all four groups (*p* = 0.003, pseudo-F = 2.578; *p* = 0.001, pseudo-F = 2.639, respectively). When performing pairwise tests for the community difference between two groups, the weighted Unifrac distance used for abundance indicated significant differences in the gut microbial community composition between all pairs of groups except for the G0 and G1 groups and for the G1 and G3 groups (see Table S2, Supplementary Materials, for full pairwise results). The unweighted Unifrac distance used for the presence/absence of ASVs was also significantly different between the G1 and G3 groups and between the G0 and G3 groups. However, due to the numerous sample numbers and interindividual variation, the fecal microbiota between the G0 and G3 groups could not be separated clearly by principal coordinates analysis (Figure S1, Supplementary Materials), although the differences in microbial community composition were significant between the two groups in all beta diversity indices.

### 3.3. Altered Gut Microbial Composition According to NAFLD Status

To better understand how the microbial community composition changed with NAFLD persistence, we investigated which organisms were present at different taxonomic levels and their relative abundance levels. Overall, 15 phyla, 31 classes, 47 orders, 86 families, 222 genera, and 328 species were detected. Bacterioidetes (49.76%), Firmicutes (43.74%), Proteobacteria (3.71%), and Actinobacteria (1.40%) were the most abundant phyla across all subjects. Other phyla identified, such as Verrucomicrobia, Fusobacteria, Tenericutes, and Cyanobacteria, had a relatively low abundance (<1%).

The ANCOM on the phylum through species levels revealed far more significant results in the comparison among the four groups after adjusting for age, sex, and BMI. Differential abundance testing with ANCOM revealed two phyla, two classes, five families, six genera, and two species that were differentially abundant among the four groups (Table 2). The phyla Fusobacteria (W = 13) and Tenericutes (W = 11) including their class Fusobacteriia (W = 27) and Mollicutes (W = 24) showed significantly different abundance levels across the four groups. The “W = 13” of Fusobacteria indicates the phylum was significantly different relative to the 13 other phyla across the four groups.

Pairwise comparisons were undertaken to identify the significant differences among the four groups. The beta diversity results were verified by pairwise ANCOM analysis and indicated specific taxa were significantly different between the G0 and G3 groups. Results from the both pairwise ACOM and MaAsLin tests indicated that G3 had a significantly lower abundance of the order RF39, the families Christensenellaceae and Odoribacteraceae, the genera *Oscillospira*, *Odoribacter*, and *Coprococcus*, and the species *eutactus* compared with the G0 group. Interestingly, the results showed that the family Christensenellaceae showed lower abundance in both the G2 and G3 groups than in the G0 and G1 groups. Additionally, *Coprococcus eutactus* had significantly lower abundance in both the G2 and G3 groups than in the G0 group. Among the significant results across all groups, only the *Bacteroides coprophilus* showed a difference between the G0 and G1 groups, but not between the G0 and G3 groups.

**Figure 4:**
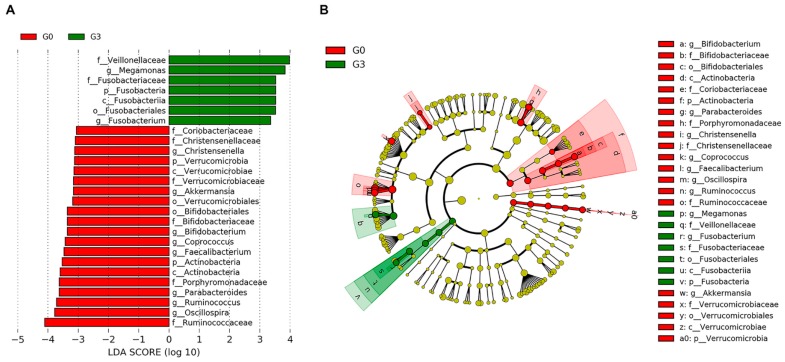
Differentially abundant bacterial taxa in fecal samples from the control (G0) and the persistent nonalcoholic fatty liver disease (NAFLD) group. (**A**) A forest plot showing the linear discriminant analysis (LDA) score (effect size) indicating significant differences in the bacterial taxa between the G0 (red) and G3 (green) groups (LDA score > 3.0; *p* < 0.05). (**B**) Cladogram generated using the LDA effect size (LEfSe) method indicating the phylogenetic distribution of microbes associated with the G0 and G3 groups.

The results revealed significant differences in the composition of gut microbiota, mainly between the G0 and G3 groups. LEfSe analysis also confirmed that the families Ruminococcaceae, Porphyromonadaceae, and Christensenellaceae were significantly enriched in the control group (G0) compared with the persistent NAFLD group (G3) (LDA > 3; *p* < 0.05, Figure 4). The two genera of the *Oscillospira* and *Ruminococcus* within Ruminococcaceae and *Coprococcus* within Lachnospiraceae also displayed significantly higher abundances in the control group than in the persistent NAFLD group.

### 3.4. Predicted Functional Microbiota in NAFLD Groups

**Figure 5:**
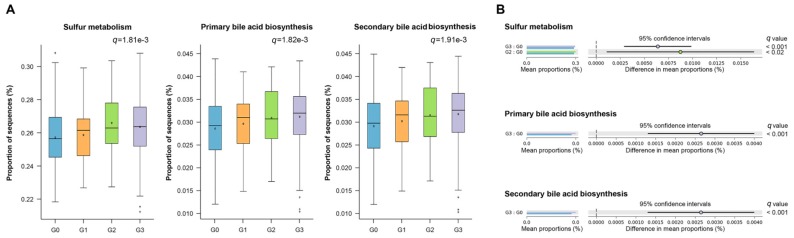
Prediction of metagenome functional content correlated with fatty liver using Phylogenetic Investigation of Communities by Reconstruction of Unobserved States (PICRUSt). (**A**) Box plot showing the distribution in the proportion of specific pathways assigned to samples from the four groups. Boxes indicate the interquartile range (IQR). The IQR is the 25th to 75th percentile. The median value is shown as a line within the box and the mean value is shown as an asterisk. Whiskers extend to the most extreme value within 1.5 × IQR. Possible outliers are shown as crosses. (**B**) Post hoc plot for each pathway indicating the difference in mean proportions for each pair of groups. The box plot (**A**) and extended error bar plot (**B**) indicate Kyoto Encyclopedia of Genes and Genomes (KEGG) pathways where two-sided Welch’s *t*-test produced a *q* < 05, adjusted using the Bonferroni method.

Based on the functionality predictions using PICRUSt and STAMP, differences in the KO composition across all groups were detected by ANOVA. Thirty-three pathways satisfied the specified filters of effect size >0.03 and *q* < 0.05 (Table S3, Supplementary Materials). Figure 5A shows the top three pathways among these 33 pathways. The sulfur metabolism pathway was significantly different across all groups (*q* = 1.81 × 10^−3^, ANOVA). Pairwise Tukey–Kramer post hoc tests indicated the pathway was highly enriched in the gut microbiota of the G2 and G3 groups compared with the G0 group (*q* < 0.02 and < 0.001, respectively; Figure 5B). Pathways related to primary bile acid biosynthesis and secondary bile acid biosynthesis were also highly detected in the G3 group compared with the G0 group (*q* < 0.001 and < 0.001, respectively).

## 4. Discussion

In this large longitudinal study, we investigated the association between the gut microbiome and persistent NAFLD. To the best of our knowledge, this is the first cohort study to demonstrate the relationship of the gut microbiota with persistent NAFLD compared with the control group without NAFLD at both baseline and follow-up. We found a lower alpha diversity and distinct compositions of gut microbiota in subjects with persistent NAFLD compared with the controls. We also characterized the association of persistent NAFLD with potentially important gut microbes that mediate energy expenditure and promote chronic inflammation.

Previous studies reported the relationship of gut microbiota with NAFLD and NASH [15,16,17,18,37], and the majority of these studies showed a decrease in the overall bacterial diversity in NAFLD. Prior studies characterizing intestinal microbiota in NASH demonstrated an association between the gut microbiome and liver health [16,18]. These studies, however, were mainly cross-sectional studies, which are limited by the temporal ambiguity between the gut microbiota and NAFLD. Furthermore, NAFLD is a reversible condition and fatty liver status can change over time and may have different prognostic implications for the risk of diabetes or CVD [9,10]. The identification of persistent NAFLD is important for predicting increased CVD risk as a surrogate marker for atherosclerosis. A longitudinal study of 16 histology-proven NASH patients demonstrated that improved intrahepatic triglyceride content, based on proton magnetic resonance spectroscopy, was associated with a reduction in the abundance of Firmicutes and increased Bacteroidetes [37]. In the present longitudinal cohort study of 766 individuals, including 238 patients with persistent NAFLD, we identified the distinctive features of the gut microbiota in patients with persistent NAFLD compared with non-NAFLD patients.

We characterized the different patterns of the gut microbiota according to NAFLD status. At the phylum level, we identified an increased abundance of Fusobacteria, including lower taxa levels, in the group with persistent NAFLD. Fusobacteriaceae includes bacterial families characterized by butyrate-producing capacity. In a previous study, NASH patients were characterized by a higher abundance of Fusobacteria and Fusobacteriaceae compared with NAFLD and healthy controls [38]. The impact of Fusobacteriaceae on metabolic diseases could be attributed to the direct or indirect activation of inflammatory signals, as well as the production of bacterial metabolites such as short-chain fatty acids (SCFAs).

In contrast, the genera *Oscillospira* and *Ruminococcus* of the family Ruminococcaceae displayed significantly lower abundance in the group with persistent NAFLD compared with the controls and patients with developed NAFLD. This finding is consistent with previous results on the gut microbiota of NASH patients, which revealed a lower abundance of the family Ruminococcaceae [16,17]. Ruminococcaceae Gram-positive bacteria, which are commonly found in the intestines of mammals, show the ability to break down cellulose and hemicellulose in plant material. These compounds are subsequently fermented and converted to SCFAs, which can be absorbed and used for energy by the host. The protective role of SCFAs against gut inflammation was well demonstrated [15,39]. The lower abundance of *Oscillospira* in NAFLD is also consistent with the previous finding that it was less abundant in NAFLD, NASH, and obese patients compared with controls [40]. We also found a lower abundance of *Coprococcus* in persistent NAFLD patients, which is a common finding in NAFLD patients [41]. A lower abundance of *Coprococcus* was reported in other inflammatory conditions [42]. Thus, the decreased abundance of *Coprococcus* may promote chronic inflammation that may contribute to the pathogenesis of NAFLD [43].

Notably, pathways related to primary and secondary bile acid biosynthesis were highly detected in the persistent NAFLD group compared with the non-NAFLD group. Several studies reported the effect of bile acids on gut microbial communities. Kakiyama et al. reported a link between liver health, bile composition, and gut microbiome structure; as an example, patients with cirrhosis exhibit lower levels of fecal bile acids, which reflects a drop in the conversion of primary to secondary bile acids [44]. This decrease may be correlated with an altered gut microbiome upon the overgrowth of potential pathogens and decreased the abundance of Gram-positive bacteria like Lachnospiraceae and Ruminococcaceae [45], which are consistent with our results. Bile acids are hormones that normally regulate hepatic cholesterol and glucose metabolisms, as well as lipid solubilization, which involves metabolic processes [46]. Changes in bile acid composition and synthesis can potentiate hepatotoxicity through pro-inflammatory mechanisms, membrane damage, or cytotoxicity [47]. The impact of the gut microbiome on the persistence of NAFLD may be mediated in part through bile acids since the gut microbiome can influence the size and composition of the bile acid pool through their effects on bile acid synthesis, deconjugation, and conversion of primary to secondary bile acids. Furthermore, bile acids may alter the composition of the gut microbiota through their detergent effects on bacterial cell membranes [48]. In a prior analysis of adults with biopsy-confirmed NAFLD, patients with NAFLD, particularly those with NASH, showed higher total and primary bile acid levels in the stool and increased bile acid synthesis in the liver that correlated with intestinal dysbiosis [49]. In another study, the composition of the gut microbiome in patients with NASH reflected the elevated secondary bile acid production, with increased taurine and glycine metabolizing bacteria in the gut of patients with NASH [50]. These findings may represent the hepatotoxic effect of primary and secondary bile acids on NAFLD and also support our observation that the composition of the gut microbiome is associated with the production of bile acids and subsequently influences the persistence of NAFLD. The reciprocal interaction between bile acids and the gut microbiota may regulate the intestinal permeability, low-grade inflammation, and immune balance and contribute to the development and persistence of NAFLD [14].

We also found that intestinal bacteria involved in the sulfur metabolism pathway were enriched in patients with persistent NAFLD. The acquisition of sulfur is important to both human and symbiotic gastrointestinal tract microbiota. The liver is an important organ in the regulation of hydrogen sulfide (H2S) metabolism, and H2S homeostasis plays a critical role in hepatic lipotoxicity [51,52]. Impaired hepatic H2S synthesis was reported in NAFLD models in vitro and in vivo and is associated with hepatic fibrosis and cirrhosis [51,53]. Further studies are needed to elucidate the role of gastrointestinal hepatic H2S in the development and progression of NAFLD.

Our study had several limitations. Firstly, we only obtained the baseline fecal samples of participants without microbiome data at follow-up, which limited our ability to examine the relationship of the change in the fecal microbiome over time with NAFLD status. Further analysis including serial data of gut microbiota in follow-up periods is required to elucidate the longitudinal association between microbiome changes and changes in NAFLD over time. Nevertheless, our findings indicate that the gut microbiota at baseline shows a potential link with the persistence of NAFLD. Secondly, NAFLD was not histologically confirmed using liver biopsies. Instead, we used the diagnosis based on US findings performed by experienced radiologists at baseline and the follow-up visits. Although US assessment has an acceptable degree of diagnostic accuracy for detecting moderate to severe fatty liver compared with histology, with a sensitivity and specificity of 84.8% and 93.6%, respectively, it cannot detect fatty infiltration below a threshold of 10–30% [54]. Thus, there is a possibility that patients with NAFLD might have been classified as those without NAFLD from the use of the abdominal US. Additionally, other information on the staging of steatosis and fibrosis was unavailable. Transient elastography, a non-invasive and reliable method for assessment of liver fibrosis, can be a reasonable modality in epidemiological research settings [55]. Unfortunately, since we used data from individuals who participated in regular health check-ups, for which transient elastography was not a routine part, data on transient elastography or liver biopsy were not available. Based on the fibrosis-4 score, a validated noninvasive fibrosis marker, no cases of fatty liver and high probability of advanced fibrosis were identified, limiting our ability to perform additional analysis in relation to hepatic fibrosis. Thirdly, this study could not find any significant association between G2 and G3 and between G0 and G1. The reason for the lack of this association can be explained by the small number of G1 (*n* = 40) and G2 (*n* = 35) participants. Future research with sufficient sample size of each group and microbiome data both at baseline and follow-up is needed to examine the impact of the gut microbiome on the development and regression of NAFLD; these may be implemented in therapeutic targets for NAFLD. Finally, our study focused on Korean men and women. Therefore, our results may not be generalizable to other populations with different ages, comorbidities, or race/ethnicity characteristics.

## 5. Conclusions

This cohort study demonstrated the influence of baseline microbiota on persistent NAFLD while considering changes in NAFLD status over time. Further studies should address the underlying mechanisms related to sulfur metabolism and bile acid biosynthesis, which may serve as a potential therapeutic target in NAFLD.

## Figures and Tables

**Table 1:** Baseline characteristics of study participants by nonalcoholic fatty liver status.

Characteristics	Overall	Nonalcoholic Fatty Liver Status			
		None (G0)	Developed (G1)	Regressed (G2)	Persistent (G3)
Number	766	453	40	35	238
Age (years) ^a^	44.9 (8.1)	44.2 (8.1)	45.1 (8.7)	44.9 (6.3)	46.2 (7.9)
Male (%)	61.2	49.7	67.5	77.1	79.8
Current smoker (%)	18.9	14.5	28.2	41.2	22.3
Alcohol intake (%) ^b^	30.8	26.7	30.8	42.9	36.6
HEPA (%)	20.8	24.5	20.0	14.3	14.7
High education level (%) ^c^	86.8	86.7	82.1	90.9	87.1
Diabetes (%)	4.6	2.4	7.5	0	8.8
Prediabetes (%) ^d^	21.5	12.8	25.0	22.9	37.4
Hypertension (%)	12.1	6.2	2.5	14.3	24.8
Metabolic syndrome (%) ^e^	15.1	3.6	3.7	11.1	31.1
BMI (kg/m^2^)	23.4 (3.0)	22.0 (2.4)	23.4 (2.2)	24.5 (2.6)	25.9 (2.7)
Waist circumference (cm) ^a^	81.7 (8.7)	77.6 (7.2)	82.5 (5.5)	84.2 (6.5)	89.2 (6.8)
Systolic BP (mmHg) ^a^	108.6 (13.3)	104.8 (12.1)	108.0 (11.9)	113.3 (11.0)	115.2 (13.3)
Diastolic BP (mmHg) ^a^	70.3 (10.1)	67.6 (9.1)	70.8 (8.9)	74.1 (9.7)	74.7 (10.5)
Glucose (mg/dL) ^a^	95.1 (15.6)	91.9 (10.7)	96.7 (17.2)	95.6 (7.6)	100.8 (21.4)
Total cholesterol (mg/dL) ^a^	196.7 (32.9)	191.0 (29.9)	205.7 (41.8)	206.1 (30.5)	204.6 (35.0)
LDL cholesterol (mg/dL) ^a^	119.3 (29.6)	112.7 (26.2)	127.8 (35.7)	127.6 (27.4)	129.2 (31.6)
Triglycerides (mg/dL) ^f^	96 (72–143)	81 (62–107)	116 (84–163)	115 (83–150)	140 (96–189)
HDL cholesterol (mg/dL) ^a^	57.0 (14.3)	61.7 (14.0)	54.7 (12.6)	55.8 (10.7)	48.8 (11.6)
Fibrosis-4 score ^a,^ ^g^	0.89 (0.35)	0.91 (0.36)	0.88 (0.29)	0.79 (0.25)	0.87 (0.33)

Data are the ^a^ mean (standard deviation) and ^f^ median (interquartile range). HEPA = health-enhancing physical activity; BMI = body mass index; BP, blood pressure; HDL, high-density lipoprotein; LDL, low-density lipoprotein. ^b^ ≥10 g/day; ^c^ ≥college graduate; ^d^ defined as fasting blood glucose ≥100 mg/dL; ^e^ defined as having three or more of the following components using the harmonized criteria [36]: (1) abdominal obesity (waist circumference ≥90 cm in men and ≥80 cm in women); (2) fasting blood glucose ≥100 mg/d or current use of blood glucose-lowering agents; (3) blood pressure ≥130/85 mmHg or current use of blood pressure-lowering agents; (4) triglyceride level ≥150 mg/dL or current use of lipid-lowering agents; and (5) HDL-cholesterol <40 mg/dL in men and HDL-cholesterol <50 mg/dL in women; ^g^ calculated as follows: Fibrosis-4 score= (age (years) × aspartate aminotransferase (AST) (U/L))/(platelet count (×10^9^/L) × alanine aminotransferase (ALT) (U/L)^1/2^).

**Table 2:** Detection of differentially abundant taxa among groups for the fatty liver persistence.

All groups (G0, G1, G2, and G3)			W ^b^ (Coefficients ^c^) from the Pairwise Groups					
Taxa Level ^a^	Taxonomic Assignment	W ^b^	G0 vs. G1	G0 vs. G2	G0 vs. G3	G1 vs. G2	G1 vs. G3	G2 vs. G3
Phylum	p_Fusobacteria	13	0	0	13 (0.013 *)	0	0	0
	p_Tenericutes	11	0	0	11 (−0.006 *)	0	9 (−0.009)	0
Class	p_Fusobacteria; c_Fusobacteriia	27	0	0	27 (0.013 *)	0	0	0
	p_Tenericutes; c_Mollicutes	24	0	0	27 (−0.006 *)	0	21 (−0.009)	0
Order	p_Fusobacteria; c_Fusobacteriia; o_Fusobacteriales	43	0	0	43 (0.013 *)	0	0	0
	p_Tenericutes; c_Mollicutes; o_RF39	42	0	0	42 (−0.007 *)	0	35 (−0.009)	0
Family	p_Fusobacteria; c_Fusobacteriia; o_Fusobacteriales; f_Fusobacteriaceae	75	0	0	76 (0.013 *)	0	0	0
	p_Firmicutes; c_Clostridia; o_Clostridiales; f_Christensenellaceae	71	0	56 (−0.008 *)	69 (−0.006 **)	60 (−0.011 *)	65 (−0.008 *)	0
	p_Bacteroidetes; c_Bacteroidia; o_Bacteroidales; f_Rikenellaceae	67	0	0	71 (−0.017)	0	0	0
	p_Bacteroidetes; c_Bacteroidia; o_Bacteroidales; f_Odoribacteraceae	67	0	0	69 (−0.006 *)	0	44 (−0.013 *)	0
	p_Bacteroidetes; c_Bacteroidia; o_Bacteroidales; f_Porphyromonadaceae	61	0	0	68 (−0.009)	0	44 (−0.018)	0
Genus	p_Firmicutes; c_Clostridia; o_Clostridiales; f_Ruminococcaceae;g_*Oscillospira*	199	0	0	205 (−0.019 *)	0	0	0
	p_Bacteroidetes; c_Bacteroidia; o_Bacteroidales; f_Odoribacteraceae; g_*Odoribacter*	195	0	0	200 (−0.010 **)	0	168 (−0.013 *)	0
	p_Fusobacteria; c_Fusobacteriia; o_Fusobacteriales; f_Fusobacteriaceae; g_*Fusobacterium*	193	0	0	196 (−0.011)	0	0	0
	p_Firmicutes; c_Clostridia; o_Clostridiales; f_Ruminococcaceae; g_*Ruminococcus*	190	0	0	200 (−0.010)	0	164 (−0.023)	0
	p_Bacteroidetes; c_Bacteroidia; o_Bacteroidales; f_Porphyromonadaceae; g_*Parabacteroides*	179	0	0	189 (−0.009)	0	153 (−0.018)	0
	p_Firmicutes; c_Clostridia; o_Clostridiales; f_Lachnospiraceae; g_*Coprococcus*	170	0	0	186 (−0.016 *)	0	0	0
Species	p_Firmicutes; c_Clostridia; o_Clostridiales; f_Lachnospiraceae; g_*Coprococcus*;s_*eutactus*	288	0	276 (−0.026 **)	290 (−0.016 **)	0	0	0
	p_Bacteroidetes; c_Bacteroidia; o_Bacteroidales; f_Bacteroidaceae; g_*Bacteroides*;s_*coprophilus*	274	251 (0.036 *)	0	21 (0.012)	0	0	0

Adjusted for age, sex, and BMI. ^a^ # of phylum: 15, # of class: 31, # of order: 47, # of family: 86, # of genera: 222, # of species: 328. ^b^ W = X for taxon k, then H0k is rejected X times. The W statistic for the significantly different taxa relative to more than 70% other taxa in each taxa level is shown in bold. p_ = phylum; c_ = class; o_ = order; f_ = family; g_ = genus; s_ = species. ^c^ The coefficients from the generalized linear model using MaAsLin on pairwise testing between two groups. * *p* < 0.05, ** *p* < 0.01.

## Supplementary Materials

The following are available online at https://www.mdpi.com/2077-0383/8/8/1089/s1: Figure S1: Beta diversity analysis between the G0 and G3 groups. (A) Weighted UniFrac and (B) Bray–Curtis PCoA plots of individual sample in each group; Table S1: Comparisons of the alpha diversity between fatty liver groups in pairwise Kruskal–Wallis test; Table S2: Beta diversity between fatty liver groups in pairwise PERMANOVA; Table S3: Predicted functional microbiota for fatty liver persistence using PICRUSt.

## References

[1] Matteoni C.A., Younossi Z.M., Gramlich T., Boparai N., Liu Y.C., McCullough A.J. (1999). Nonalcoholic fatty liver disease: A spectrum of clinical and pathological severity. Gastroenterology.

[2] Rawla P., Sunkara T., Muralidharan P., Raj J.P. (2018). Update in global trends and aetiology of hepatocellular carcinoma. Contemp. Oncol. (Pozn).

[3] Chalasani N., Younossi Z., Lavine J.E., Diehl A.M., Brunt E.M., Cusi K., Charlton M., Sanyal A.J. (2012). The diagnosis and management of non-alcoholic fatty liver disease: practice Guideline by the American Association for the Study of Liver Diseases, American College of Gastroenterology, and the American Gastroenterological Association. Hepatology.

[4] Younossi Z.M., Koenig A.B., Abdelatif D., Fazel Y., Henry L., Wymer M. (2016). Global epidemiology of nonalcoholic fatty liver disease-Meta-analytic assessment of prevalence, incidence, and outcomes. Hepatology.

[5] Kim D., Siddique O., Perumpail B.J., Ahmed A., Wong R.J., Gish R.G. (2019). Clinical epidemiology of NAFLD. Clinical Epidemiology of Chronic Liver Diseases.

[6] Musso G., Gambino R., Cassader M., Pagano G. (2011). Meta-analysis: Natural history of non-alcoholic fatty liver disease (NAFLD) and diagnostic accuracy of non-invasive tests for liver disease severity. Ann. Med..

[7] Anstee Q.M., Targher G., Day C.P. (2013). Progression of NAFLD to diabetes mellitus, cardiovascular disease or cirrhosis. Nat. Rev. Gastroenterol. Hepatol..

[8] Yki-Jarvinen H. (2014). Non-alcoholic fatty liver disease as a cause and a consequence of metabolic syndrome. Lancet Diabetes Endocrinol..

[9] Kim D., Chung G.E., Kwak M.S., Seo H.B., Kang J.H., Kim W., Kim Y.J., Yoon J.H., Lee H.S., Kim C.Y. (2016). Body fat distribution and risk of incident and regressed nonalcoholic fatty liver disease. Clin. Gastroenterol. Hepatol..

[10] Sung K.C., Wild S.H., Byrne C.D. (2013). Resolution of fatty liver and risk of incident diabetes. J. Clin. Endocrinol. Metab..

[11] Sinn D.H., Cho S.J., Gu S., Seong D., Kang D., Kim H., Yi B.K., Paik S.W., Guallar E., Cho J. (2016). Persistent nonalcoholic fatty liver disease increases risk for carotid atherosclerosis. Gastroenterology.

[12] Bae J.C., Han J.M., Cho J.H., Kwon H., Park S.E., Park C.Y., Lee W.Y., Oh K.W., Kwon S., Park S.W. (2018). The persistence of fatty liver has a differential impact on the development of diabetes: The Kangbuk Samsung Health Study. Diabetes Res. Clin. Pract..

[13] Sunkara T., Rawla P., Ofosu A., Gaduputi V. (2018). Fecal microbiota transplant—A new frontier in inflammatory bowel disease. J. Inflamm. Res..

[14] Aron-Wisnewsky J., Gaborit B., Dutour A., Clement K. (2013). Gut microbiota and non-alcoholic fatty liver disease: New insights. Clin. Microbiol. Infect..

[15] Jiang W., Wu N., Wang X., Chi Y., Zhang Y., Qiu X., Hu Y., Li J., Liu Y. (2015). Dysbiosis gut microbiota associated with inflammation and impaired mucosal immune function in intestine of humans with non-alcoholic fatty liver disease. Sci. Rep..

[16] Zhu L., Baker S.S., Gill C., Liu W., Alkhouri R., Baker R.D., Gill S.R. (2013). Characterization of gut microbiomes in nonalcoholic steatohepatitis (NASH) patients: A connection between endogenous alcohol and NASH. Hepatology.

[17] Raman M., Ahmed I., Gillevet P.M., Probert C.S., Ratcliffe N.M., Smith S., Greenwood R., Sikaroodi M., Lam V., Crotty P. (2013). Fecal microbiome and volatile organic compound metabolome in obese humans with nonalcoholic fatty liver disease. Clin. Gastroenterol. Hepatol..

[18] Mouzaki M., Comelli E.M., Arendt B.M., Bonengel J., Fung S.K., Fischer S.E., McGilvray I.D., Allard J.P. (2013). Intestinal microbiota in patients with nonalcoholic fatty liver disease. Hepatology.

[19] Chang Y., Cho Y.K., Kim Y., Sung E., Ahn J., Jung H.S., Yun K.E., Shin H., Ryu S. (2019). Nonheavy DRINKING and worsening of noninvasive fibrosis markers in nonalcoholic fatty liver disease: A cohort study. Hepatology.

[20] Park J.T., Kim B.G., Jhun H.J. (2008). Alcohol consumption and the CAGE questionnaire in Korean adults: results from the Second Korea National Health and Nutrition Examination Survey. J. Korean Med. Sci..

[21] Craig C.L., Marshall A.L., Sjostrom M., Bauman A.E., Booth M.L., Ainsworth B.E., Pratt M., Ekelund U., Yngve A., Sallis J.F. (2003). International physical activity questionnaire: 12-country reliability and validity. Med. Sci. Sports Exerc..

[22] World Health Organization, Regional office for the western pacific (2000). The Asia-Pacific Perspective: Redefining Obesity and Its Treatment.

[23] Mathiesen U.L., Franzen L.E., Aselius H., Resjo M., Jacobsson L., Foberg U., Fryden A., Bodemar G. (2002). Increased liver echogenicity at ultrasound examination reflects degree of steatosis but not of fibrosis in asymptomatic patients with mild/moderate abnormalities of liver transaminases. Dig. Liver Dis..

[24] Kim H.N., Yun Y., Ryu S., Chang Y., Kwon M.J., Cho J., Shin H., Kim H.L. (2018). Correlation between gut microbiota and personality in adults: A cross-sectional study. Brain. Behav. Immun..

[25] Kozich J.J., Westcott S.L., Baxter N.T., Highlander S.K., Schloss P.D. (2013). Development of a dual-index sequencing strategy and curation pipeline for analyzing amplicon sequence data on the MiSeq Illumina sequencing platform. Appl. Environ. Microbiol..

[26] Callahan B.J., McMurdie P.J., Rosen M.J., Han A.W., Johnson A.J., Holmes S.P. (2016). DADA2: High-resolution sample inference from Illumina amplicon data. Nat. Methods.

[27] Bolyen E., Rideout J., Dillon M., Bokulich N., Abnet C., Al-Ghalith G., Alexander H., Alm E., Arumugam M., Asnicar F. (2018). QIIME 2: Reproducible, interactive, scalable, and extensible microbiome data science. PeerJ Prepr.

[28] Faith D.P., Baker A.M. (2006). Phylogenetic diversity (PD) and biodiversity conservation: Some bioinformatics challenges. Evol. Bioinform. Online.

[29] Bray J.R., Curtis J.T. (1957). An ordination of the upland forest communities of Southern Wisconsin. Ecol. Monogr..

[30] Lozupone C., Lladser M.E., Knights D., Stombaugh J., Knight R. (2011). UniFrac: An effective distance metric for microbial community comparison. ISME J..

[31] Mandal S., Van Treuren W., White R.A., Eggesbo M., Knight R., Peddada S.D. (2015). Analysis of composition of microbiomes: A novel method for studying microbial composition. Microb. Ecol. Health Dis..

[32] Morgan X.C., Tickle T.L., Sokol H., Gevers D., Devaney K.L., Ward D.V., Reyes J.A., Shah S.A., LeLeiko N., Snapper S.B. (2012). Dysfunction of the intestinal microbiome in inflammatory bowel disease and treatment. Genome Biol..

[33] Segata N., Izard J., Waldron L., Gevers D., Miropolsky L., Garrett W.S., Huttenhower C. (2011). Metagenomic biomarker discovery and explanation. Genome Biol..

[34] Langille M.G., Zaneveld J., Caporaso J.G., McDonald D., Knights D., Reyes J.A., Clemente J.C., Burkepile D.E., Vega Thurber R.L., Knight R. (2013). Predictive functional profiling of microbial communities using 16S rRNA marker gene sequences. Nat. Biotechnol..

[35] Parks D.H., Tyson G.W., Hugenholtz P., Beiko R.G. (2014). STAMP: Statistical analysis of taxonomic and functional profiles. Bioinformatics.

[36] Alberti K.G., Eckel R.H., Grundy S.M., Zimmet P.Z., Cleeman J.I., Donato K.A., Fruchart J.C., James W.P., Loria C.M., Smith S.C. (2009). Harmonizing the metabolic syndrome: A joint interim statement of the international diabetes federation task force on epidemiology and prevention; National heart, lung, and blood institute; American heart association; World heart federation; International atherosclerosis society; and international association for the study of obesity. Circulation.

[37] Wong V.W., Tse C.H., Lam T.T., Wong G.L., Chim A.M., Chu W.C., Yeung D.K., Law P.T., Kwan H.S., Yu J. (2013). Molecular characterization of the fecal microbiota in patients with nonalcoholic steatohepatitis-a longitudinal study. PLoS ONE.

[38] Rau M., Rehman A., Dittrich M., Groen A.K., Hermanns H.M., Seyfried F., Beyersdorf N., Dandekar T., Rosenstiel P., Geier A. (2018). Fecal SCFAs and SCFA-producing bacteria in gut microbiome of human NAFLD as a putative link to systemic T-cell activation and advanced disease. United Eur. Gastroenterol. J..

[39] Kles K.A., Chang E.B. (2006). Short-chain fatty acids impact on intestinal adaptation, inflammation, carcinoma, and failure. Gastroenterology.

[40] Del Chierico F., Nobili V., Vernocchi P., Russo A., Stefanis C., Gnani D., Furlanello C., Zandona A., Paci P., Capuani G. (2017). Gut microbiota profiling of pediatric nonalcoholic fatty liver disease and obese patients unveiled by an integrated meta-omics-based approach. Hepatology.

[41] Sharpton S.R., Ajmera V., Loomba R. (2019). Emerging Role of the Gut Microbiome in Nonalcoholic Fatty Liver Disease: From Composition to Function. Clin. Gastroenterol. Hepatol..

[42] Shaw K.A., Bertha M., Hofmekler T., Chopra P., Vatanen T., Srivatsa A., Prince J., Kumar A., Sauer C., Zwick M.E. (2016). Dysbiosis, inflammation, and response to treatment: A longitudinal study of pediatric subjects with newly diagnosed inflammatory bowel disease. Genome Med..

[43] Da Silva H.E., Teterina A., Comelli E.M., Taibi A., Arendt B.M., Fischer S.E., Lou W., Allard J.P. (2018). Nonalcoholic fatty liver disease is associated with dysbiosis independent of body mass index and insulin resistance. Sci. Rep..

[44] Kakiyama G., Pandak W.M., Gillevet P.M., Hylemon P.B., Heuman D.M., Daita K., Takei H., Muto A., Nittono H., Ridlon J.M. (2013). Modulation of the fecal bile acid profile by gut microbiota in cirrhosis. J. Hepatol..

[45] Urdaneta V., Casadesus J. (2017). Interactions between Bacteria and Bile Salts in the Gastrointestinal and Hepatobiliary Tracts. Front. Med. (Lausanne).

[46] Staels B., Fonseca V.A. (2009). Bile acids and metabolic regulation: Mechanisms and clinical responses to bile acid sequestration. Diabetes Care.

[47] Allen K., Jaeschke H., Copple B.L. (2011). Bile acids induce inflammatory genes in hepatocytes: A novel mechanism of inflammation during obstructive cholestasis. Am. J. Pathol..

[48] Begley M., Gahan C.G., Hill C. (2005). The interaction between bacteria and bile. FEMS Microbiol. Rev..

[49] Mouzaki M., Wang A.Y., Bandsma R., Comelli E.M., Arendt B.M., Zhang L., Fung S., Fischer S.E., McGilvray I.G., Allard J.P. (2016). Bile acids and dysbiosis in Non-Alcoholic fatty liver disease. PLoS ONE.

[50] Jiao N., Baker S.S., Chapa-Rodriguez A., Liu W., Nugent C.A., Tsompana M., Mastrandrea L., Buck M.J., Baker R.D., Genco R.J. (2018). Suppressed hepatic bile acid signalling despite elevated production of primary and secondary bile acids in NAFLD. Gut.

[51] Li M., Xu C., Shi J., Ding J., Wan X., Chen D., Gao J., Li C., Zhang J., Lin Y. (2018). Fatty acids promote fatty liver disease via the dysregulation of 3-mercaptopyruvate sulfurtransferase/hydrogen sulfide pathway. Gut.

[52] Mani S., Cao W., Wu L., Wang R. (2014). Hydrogen sulfide and the liver. Nitric Oxide.

[53] Fiorucci S., Antonelli E., Mencarelli A., Orlandi S., Renga B., Rizzo G., Distrutti E., Shah V., Morelli A. (2005). The third gas: H2S regulates perfusion pressure in both the isolated and perfused normal rat liver and in cirrhosis. Hepatology.

[54] Hernaez R., Lazo M., Bonekamp S., Kamel I., Brancati F.L., Guallar E., Clark J.M. (2011). Diagnostic accuracy and reliability of ultrasonography for the detection of fatty liver: A meta-analysis. Hepatology.

[55] Castera L., Forns X., Alberti A. (2008). Non-invasive evaluation of liver fibrosis using transient elastography. J. Hepatol..

