# Supplementary material for: Gut Microbiota and Risk of Persistent Nonalcoholic Fatty Liver Diseases
Source: J Clin Med. 2019 Jul 24;8(8):1089. doi: 10.3390/jcm8081089 (PMC6722749; doi:10.3390/jcm8081089)
Supplement: Supplementary file 1 [file jcm-08-01089-s001.pdf]

Table S1. Comparisons of the alpha diversity between fatty liver groups in pairwise Kruskal-Wallis test

**Alpha diversity index**	**Group**	**Group**	**H**	***p-value***	***q-value***
Observed ASVs[a]	G0 (n=453)	G1 (n=40)	1.05	3.1.E-01	3.7.E-01
	G0 (n=453)	G2 (n=35)	2.05	1.5.E-01	2.3.E-01
	G0 (n=453)	G3 (n=238)	11.77	6.0.E-04	3.6.E-03**
	G1 (n=40)	G2 (n=35)	2.92	8.7.E-02	1.7.E-01
	G1 (n=40)	G3 (n=238)	5.47	1.9.E-02	5.8.E-02
	G2 (n=35)	G3 (n=238)	0.01	9.2.E-01	9.2.E-01
Faith's PD[a]	G0 (n=453)	G1 (n=40)	1.33	2.5.E-01	3.0.E-01
	G0 (n=453)	G2 (n=35)	3.19	7.4.E-02	1.1.E-01
	G0 (n=453)	G3 (n=238)	15.05	1.0.E-04	6.3.E-04**
	G1 (n=40)	G2 (n=35)	3.82	5.1.E-02	1.0.E-01
	G1 (n=40)	G3 (n=238)	6.22	1.3.E-02	3.8.E-02*
	G2 (n=35)	G3 (n=238)	0	9.8.E-01	9.8.E-01
Shannon[a]	G0 (n=453)	G1 (n=40)	0.58	4.5.E-01	5.4.E-01
	G0 (n=453)	G2 (n=35)	4.06	4.4.E-02	8.8.E-02
	G0 (n=453)	G3 (n=238)	19.94	8.0.E-06	4.8.E-05**
	G1 (n=40)	G2 (n=35)	3.26	7.1.E-02	1.1.E-01
	G1 (n=40)	G3 (n=238)	6.21	1.3.E-02	3.8.E-02*
	G2 (n=35)	G3 (n=238)	0.01	9.1.E-01	9.1.E-01

[a]The p-values among all groups were estimated using Kruskal-Wallis test. The H=14.87, 19.06, and 23.50; p-value=1.9×10-3, 2.7×10-4, and 3.2×10-5 for the observed ASVs, Faith's PD, and Shannon, respectively.

*$q < 0.05$; **$q < 0.01$

Table S2. Beta diversity between fatty liver groups in pairwise PERMANOVA

Beta-diversity index	Group	Group	Sample size	Permutations	pseudo-F	*p-value*	*q-value*
Unweighted UniFrac distance[a]	G0	G1	493	999	0.99	4.0.E-01	4.7.E-01
	G0	G2	488	999	1.48	6.9.E-02	1.0.E-01
	G0	G3	691	999	5.5	1.0.E-03	6.0.E-03**
	G1	G2	75	999	1.67	5.8.E-02	1.0.E-01
	G1	G3	278	999	2.41	1.2.E-02	3.6.E-02*
	G2	G3	273	999	0.94	4.7.E-01	4.7.E-01
Weighted UniFrac distance[a]	G0	G1	493	999	0.42	8.4.E-01	8.4.E-01
	G0	G2	488	999	4.61	6.0.E-03	2.9.E-02*
	G0	G3	691	999	3.18	1.9.E-02	2.9.E-02*
	G1	G2	75	999	3.2	1.5.E-02	2.9.E-02*
	G1	G3	278	999	0.5	7.5.E-01	8.4.E-01
	G2	G3	273	999	3.37	1.6.E-02	2.9.E-02*
Bray-Curtis dissimilarity[a]	G0	G1	493	999	0.82	8.8.E-01	8.8.E-01
	G0	G2	488	999	1.03	3.9.E-01	7.6.E-01
	G0	G3	691	999	1.98	1.0.E-03	6.0.E-03**
	G1	G2	75	999	0.94	6.3.E-01	7.6.E-01
	G1	G3	278	999	0.95	5.5.E-01	7.6.E-01
	G2	G3	273	999	0.99	4.6.E-01	7.6.E-01

[a]The p-values among all groups were estimated using permutational multivariate analysis of variance (PERMANOVA). The psuedo-F=2.64, 2.58, and 1.26; p-value=0.001, 0.003, and 0.01 for the unweighted UniFrac, weighted UniFrac and Bray-Curtis indices, respectively.

*$q < 0.05$; **$q < 0.01$

Table S3. Predicted functional microbiota for fatty liver persistence using PICRUSt

Level_1	Level_2	Level_3	Effect size (Eta-squared)	*p-values*	*q-values* (Bonferroni)
Metabolism	Energy Metabolism	Sulfur metabolism	0.035	5.51E-06	1.81.E-03
Metabolism	Lipid Metabolism	Primary bile acid biosynthesis	0.035	5.55E-06	1.82.E-03
Metabolism	Lipid Metabolism	Secondary bile acid biosynthesis	0.035	5.82E-06	1.91.E-03
Human Diseases	Infectious Diseases	Epithelial cell signaling in Helicobacter pylori infection	0.034	8.36E-06	2.74.E-03
Cellular Processes and Signaling	Other ion-coupled transporters	Other ion-coupled transporters_Unclassified	0.034	9.38E-06	3.08.E-03
Cellular Processes and Signaling	Other transporters	Other transporters_Unclassified	0.034	9.38E-06	3.08.E-03
Genetic Information Processing	Replication and Repair	Non-homologous end-joining	0.033	1.29E-05	4.25.E-03
Cellular Processes	Cell Growth and Death	Meiosis - yeast	0.032	1.56E-05	5.11.E-03
Metabolism	Xenobiotics Biodegradation and Metabolism	Chloroalkane and chloroalkene degradation	0.032	1.76E-05	5.79.E-03
Metabolism	Energy metabolism	Energy metabolism_Unclassified	0.032	1.86E-05	6.10.E-03
Genetic Information Processing	Replication and Repair	DNA replication proteins	0.032	1.90E-05	6.24.E-03
Metabolism	Enzyme Families	Peptidases	0.032	1.91E-05	6.28.E-03
Metabolism	Amino Acid Metabolism	Amino acid related enzymes	0.032	1.95E-05	6.39.E-03
Metabolism	Metabolism of Cofactors and Vitamins	Thiamine metabolism	0.031	2.11E-05	6.91.E-03
Metabolism	Metabolism of Other Amino Acids	Selenocompound metabolism	0.031	2.19E-05	7.18.E-03
Human Diseases	Immune System Diseases	Primary immunodeficiency	0.031	2.40E-05	7.89.E-03
Metabolism	Xenobiotics Biodegradation and Metabolism	Benzoate degradation	0.031	2.44E-05	8.00.E-03
Organismal Systems	Nervous System	Glutamatergic synapse	0.031	2.46E-05	8.08.E-03
Genetic Information Processing	Replication and Repair	Homologous recombination	0.031	2.56E-05	8.41.E-03
Human Diseases	Infectious Diseases	Amoebiasis	0.031	2.57E-05	8.43.E-03
Genetic Information Processing	Translation	Ribosome biogenesis in eukaryotes	0.031	2.66E-05	8.74.E-03
Metabolism	Carbohydrate Metabolism	Propanoate metabolism	0.031	2.72E-05	8.93.E-03
Genetic Information Processing	Replication and Repair	DNA repair and recombination proteins	0.031	2.72E-05	8.93.E-03

Metabolism	Metabolism of Other Amino Acids	D-Arginine and D-ornithine metabolism	0.031	2.88E-05	9.46.E-03
Metabolism	Xenobiotics Biodegradation and Metabolism	Chlorocyclohexane and chlorobenzene degradation	0.030	3.15E-05	1.03.E-02
Metabolism	Carbohydrate Metabolism	Starch and sucrose metabolism	0.030	3.24E-05	1.06.E-02
Human Diseases	Infectious Diseases	Influenza A	0.030	3.46E-05	1.13.E-02
Cellular Processes	Cell Growth and Death	p53 signaling pathway	0.030	3.46E-05	1.14.E-02
Human Diseases	Cancers	Colorectal cancer	0.030	3.46E-05	1.14.E-02
Human Diseases	Cardiovascular Diseases	Viral myocarditis	0.030	3.46E-05	1.14.E-02
Human Diseases	Cancers	Small cell lung cancer	0.030	3.46E-05	1.14.E-02
Human Diseases	Infectious Diseases	Toxoplasmosis	0.030	3.46E-05	1.14.E-02
Genetic Information Processing	Replication and Repair	DNA replication	0.030	3.61E-05	1.18.E-02

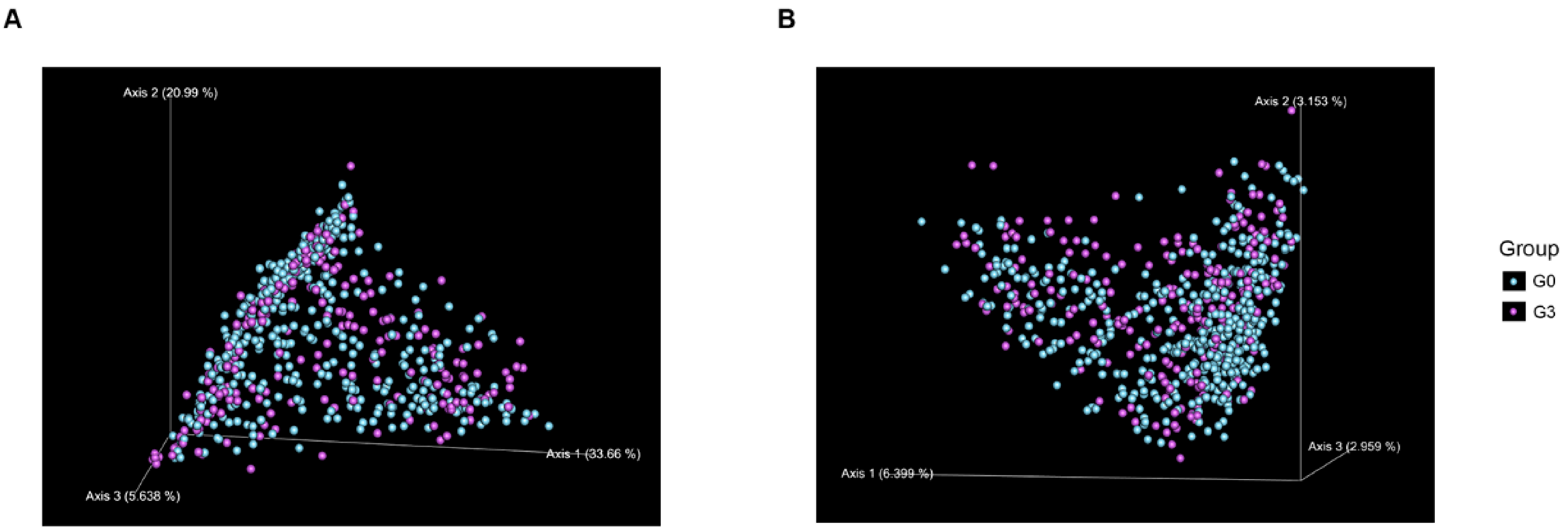

Figure S1: Beta diversity analysis between the G0 and G3 groups. **(A)** Weighted UniFrac and **(B)** Bray-Curtis PCoA plots of individual sample in each group
